# A Bayesian Approach for Sensor Optimisation in Impact Identification

**DOI:** 10.3390/ma9110946

**Published:** 2016-11-22

**Authors:** Vincenzo Mallardo, Zahra Sharif Khodaei, Ferri M. H. Aliabadi

**Affiliations:** 1 Department of Architecture, University of Ferrara, Via Quartieri 8, 44121 Ferrara, Italy; 2 Department of Aeronautics, Imperial College London, London SW7 2AZ, UK; z.sharif-khodaei@imperial.ac.uk (Z.S.K.); m.h.aliabadi@imperial.ac.uk (F.M.H.A.)

**Keywords:** probability of detection, sensor malfunctioning, genetic algorithm, non-linear finite element method, artificial neural network, structural health monitoring

## Abstract

This paper presents a Bayesian approach for optimizing the position of sensors aimed at impact identification in composite structures under operational conditions. The uncertainty in the sensor data has been represented by statistical distributions of the recorded signals. An optimisation strategy based on the genetic algorithm is proposed to find the best sensor combination aimed at locating impacts on composite structures. A Bayesian-based objective function is adopted in the optimisation procedure as an indicator of the performance of meta-models developed for different sensor combinations to locate various impact events. To represent a real structure under operational load and to increase the reliability of the Structural Health Monitoring (SHM) system, the probability of malfunctioning sensors is included in the optimisation. The reliability and the robustness of the procedure is tested with experimental and numerical examples. Finally, the proposed optimisation algorithm is applied to a composite stiffened panel for both the uniform and non-uniform probability of impact occurrence.

## 1. Introduction

The impact detection and identification strategy for existing structures is of primary importance both in structural health monitoring (SHM) and in non-destructive evaluation (NDE) techniques. Accurately detecting and characterizing an impact event based on sensor data leads us towards condition-based monitoring (CBM), where the subsequent damage can then be detected through active sensing strategies [1,2,3,4,5,6,7]. Impact identification procedures have been investigated in the last two decades, both numerically and experimentally [8,9,10,11,12,13,14]. Many of the impact localization methods use the triangulation technique based on the time of flight (ToF) of the waves generated due to an impact and captured by sensors. This approach is limited by the assumption that wave velocity must be known and remain the same in all directions. However, this is not the case for anisotropic and inhomogeneous materials. Moreover, the flexural wave velocity is not constant, and it is a function of the signal frequency, which depends on the impact mass and velocity. Methodologies applicable to anisotropic structures can be grouped into three techniques:

- Build meta-models between the sensor response and the impacts by using an inverse analysis. Such an example is the time reversal method [8]; however, once the response of the structure becomes non-linear (presence of damage), the method is not applicable. To account for the non-linearity of the problem, the genetic algorithm (GA) and artificial neural networks (ANN) have been proposed. In [9,10], ANNs were established, using a large dataset of various impact scenarios obtained numerically, to predict impact location and impact force distribution
- Having a distributed sensor array in the structure and using the hyperbolic positioning algorithm. In [11], Ciampa et al. proposed a strategy to locate impact in composite plates with six piezoelectric (PZT) sensors by calculating the time difference between each sensor. However, this techniques require an accurate estimate of the wave velocity, which is only achieved by placing the sensors in optimized locations. This might be an issue for built-up structures
- Sensor rosettes are used to estimate the wave propagation direction. In [14], Zhang et al. has used a metal-core piezoelectric fibre (MPF) sensor rosette to detect and localize impacts on metallic plates. A new procedure proposed by Zamorano et al. [13] uses groups of closely-spaced embedded sensors to calculate the angle of the impact. The method was validated for high velocity impact where the impactor pierces the composite panel. In a recent study, Qiu et al. [15] have adopted a spatial-wavenumber filtering technique with a dense 2D cross-shaped linear array of piezoelectric sensors to detect the impact direction and the distance relative to the sensor array. The validation results on composite structures show that the impact direction can be estimated accurately, but the accuracy of the impact location estimation is limited by the error in the group velocity

A key parameter in the reliability of any impact detection algorithm is the optimal number and location of sensors (see the review in [16]). Most of the impact detection and identification research is carried out in a controlled laboratory environment. However, in real structures under operational conditions, there will be many factors influencing the recorded signals. In general, uncertainties can be caused by random and systematic errors, and it is important to consider them in the design and optimisation of any SHM system for the application to real structures. The basic idea of the Bayesian approach is to treat some variables, denoted by the vector ***θ***, as random variables with joint distribution p(θ). It aims at calculating the posterior (updated) distributions of uncertain parameters for a given set of measured data. Such an approach has been often adopted in SHM techniques in the damage identification framework [17,18].

A probabilistic framework for acoustic emission (AE) source localization with PZT sensors in plate-like structures is developed in [19] and validated on an aluminium plate. The ToF and the wave velocity are considered as mutually-independent Gaussian random variables. The continuous wavelet transform (CWT) is applied to the ToF measurements, and the resulting variances in time domain and in frequency domain are correlated to the uncertainty in arrival time and in group velocity, respectively, by invoking the Heisenberg uncertainty. The extended kalman filter (EKF) is used to estimate the AE source location and the wave velocity provided that the ToF measurements and the previous variances are given.

An early contribution to the optimal sensor placement problem for damage detection laying out a theoretical framework rooted in Bayes risk is provided in [20]. The general form of the Bayes risk is first investigated and then minimized in order to provide the optimal detector, i.e., the sensor placement that either maximizes the probability of detection (PoD) or minimizes the false alarm rate. In [21], Vanli et al. proposed an optimal sensor placement strategy for damage detection to account for the non-uniform likelihood of damages on the structure.

The Bayesian-based optimisation approaches have been developed for active sensing. However, there is little to no evidence of their application to passive sensing. There still remains a challenge for applying the developed methodologies and algorithms to real structures. For example, taking the Airbus A320 family, each zone of the aircraft has a different global percentage of impact, which should be considered when optimizing the sensor locations for a real structure; see Table 1. Another challenge is to include sensor failures in the reliability analysis of the SHM system.

The main goal of the present paper is to propose a Bayesian-based sensor optimisation strategy aimed at impact identification under varying operational conditions, such as variabilities in the sensor data, sensor failure and the non-uniform probability of impact occurrence. To the authors’ best knowledge, this is the first contribution that encompasses all of the above uncertainties. The passive sensing procedure is based on a meta-modelling technique developed from a large library of impact scenarios on a composite stiffened panel as presented in [23].

The layout of the paper is as follows: The application of the Bayesian approach to the optimisation framework is detailed in Section 2 by investigating different sources of uncertainties. The impact detection algorithm is outlined in Section 2.1 along with details on the adopted artificial neural network strategy. A novel procedure is introduced and implemented in Section 2.2 in order to include the sensor malfunctioning in the impact identification algorithm. The optimisation process is carried out both excluding and including the probability of sensor malfunctioning, in Section 2.3 and Section 2.4, respectively. The computation of the probability of detection, which is necessary to define the objective function of the optimisation process, is detailed in Section 2.5 by improving the procedure outlined in [23]. The fitness function and the uncertainty inclusion are validated by experimental and numerical examples in Section 3 to assess the efficiency and robustness of the proposed procedure. Finally, Section 4 presents the application of sensor location optimisation on a composite stiffened panel.

## 2. A Bayesian Sensor Optimisation Algorithm

Here, a Bayesian framework that optimises the sensors’ position in order to maximize the probability of detection of an impact event in the presence of the probability of sensor malfunctioning and variations in the recorded data is described. Two main uncertainties are considered: (i) the error due to factors, such as instrumentation noise, bonding quality, temperature changes, loading and numerical simulation, which may affect the sensor data; (ii) the possible malfunctioning of one or more sensors, which may have failed (damaged or debonded).

The proposed procedure assumes a plate-like structure instrumented with a permanent network of transducers for recording elastic waves generated by an impact event. Each transducer works as a sensor, and it can provide the time of arrival (ToA) of the wave for a given impact. From the knowledge of the ToA from a network of sensors, it is possible to estimate the location of the impact (see Section 2.1 for a brief overview of the passive sensing methodology).

In order to compute the optimal sensor combination for a given number of sensors, a minimization process needs to be carried out. The optimisation problem is solved by the application of the genetic algorithm as developed in [23]. The procedure is implemented in the MATLAB program. Individual integer representation, scores and selection, crossover and mutation are taken from [23]. The main improvements of the code are related to the computation of the fitness function, i.e., functions $J(e_{T})$ and $J(e_{T},M_{s})$, which will be thoroughly described in this section.

### 2.1. Impact Detection Algorithm

The passive sensing algorithm used in this work for impact detection is based on the ANN technique developed in [9,23] to detect the location of an impact from sensor readings. ANN is a machine learning algorithm that uses a set of training data to build a non-linear function relating a set of input data to output data. It adapts its structure (weights) based on the input and output of the system during the learning phase. The input, in this case, is sensor signals recorded during an impact event, and the output is given by the *x* and *y* coordinates of the impact location. To reach a regularization, the ANN needs to be trained with a wide range of data, i.e., impact scenarios representing probable impact cases for the structure under analysis. It is unrealistic to assume that all of the required data can be obtained experimentally due to the large number of possible impacts. Therefore, in this work, a vast library of impact scenarios (different locations and energies), which are likely to occur during the life-time of an aircraft fuselage, were obtained by performing impact simulations on a composite stiffened panel using a validated non-linear Finite Element (FE) model. The impacts cover all of the possible locations, i.e., mid-bay, under/over the stringer and on the foot of the stringer. In addition, different types of impacts (large mass and small mass) are considered from both inside and outside of the panel. Details of the numerical model and the development of meta models can be found in [9]. The recorded sensor signals, in their discrete form, should not be used as inputs to the ANN because they contain too much information. When there are too many input parameters in the model compared to the number of training patterns, there will be the problem of overfitting, and the meta-model will not reach a converged solution. Therefore, feature extraction is applied to the sensor data. The feature used in this work as input to the ANN is the ToA of each sensor signal. The features extracted from each signal are weighted and passed into the input layer, which is connected to the hidden layer and the output layer through the non-linear activation function. The novelty of the current work is the inclusion of uncertainties in the input data (ToA) in a statistical framework and the addition of the probability of sensor malfunctioning in the ANN generation. It must be pointed out that the proposed optimisation approach is independent of the impact identification algorithm. It can be implemented with any impact identification procedure, using either experimental or numerical data.

### 2.2. Inclusion of Sensor Malfunctioning into Impact Detection

In this section, a procedure to include the malfunctioning sensor in the impact identification algorithm is presented. The way its probability of occurrence is included in the optimisation process will be discussed in the Section 2.4.

The monitored structures are usually subjected to various external load and environmental conditions that may adversely affect the functionality of the SHM sensors. Impacts may also be responsible for sensor failures. Because piezoceramic materials are brittle, sensor fracture and subsequent degradation of mechanical/electrical properties are the most common types of sensor failures. In addition, the integrity of the bonding between a PZT sensor and a host structure should be maintained and monitored throughout its service life. A completely broken sensor (hard fault) can be identified when the sensor does not produce any measurable output. However, if the sensor or the bonding is partially damaged or deteriorated, the distorted signal can potentially lead to a false indication of impact (soft fault).

There are established techniques to determine faulty sensors prior to any acquisition; see, for instance [24,25,26,27,28]. The degradation of the PZT sensor may also be detected by measuring the change in the imaginary part of the electrical admittance [7,29]. In this work, the probability that one or more sensors may be malfunctioning during the structural monitoring is included in the optimisation process. Sensor fault malfunctioning (either soft or hard) is included in the optimisation procedure by removing the ToA of the faulty sensor from the analysis. The reasoning for this approach is to increase the reliability of the detection by having more than one trained ANN for any given sensor combination.

The optimisation can be carried out using either experimental data, or numerical data, or a combination of both. The iterative sensor combination can be tested by building its representative ANN together with all of the possible ANNs obtained from the pristine sensors by removing $M_{s}$ sensors, where $M_{s}$ provides the pre-defined number of possible faulty sensors. In such an alternative, a known number of ANNs needs to be generated for each sensor combination involved in the optimisation process. If CombSens is a matrix including all of the possible sensor combinations generated from the original one by removing the faulty sensors, the procedure can be written as:

$$\begin{array}{c} for\;\;i=1:size(CombSens,1)\\ \;\%\;sensor=\text{the vector collecting the sensor combination under analysis}\\ \;a=ismember(sensor,CombSens(i,:))\\ \;b=find(a==0)\\ \;\%ToA=\text{ToA at the sensors for all the impacts}\\ \;ToA(b,:)=[]\\ \;\%\;\text{Generate the ANN without faulted sensors}\\ \;[testInd,ANN\{i\}]=ANNgenerator(ToA)\\ end \end{array}$$

The faulty sensors are selected following the probability distribution $p(M_{s})$. Two different sub-options have been considered here. In the former (Option A), exactly $M_{s}$ faulty sensors are envisaged. This means that $\frac{n!}{M_{s}!\left(n-M_{s}\right)!}$ ANNs need to be built for each sensor combination to be tested in the optimisation process:

(1) $$\begin{array}{c} nsensors=length(sensor)\\ CombSens=nchoosek(sensor,nsensors-M_{s}) \end{array}$$

In the latter (Option B), $M_{s}$ indicates the maximum number of probable faulty sensors, i.e., the ANNs to be generated are all of the ANNs extracted from the sensor combination under analysis by removing all of the *k* faulty sensors with *k* going from one to $M_{s}$:

$$\begin{array}{c} ii=0\\ for\;\;i=1:M_{s}\\ \;temp=nchoosek(sensor,nsensors-i);\\ \;CombSens(ii+1:ii+size(temp,1),1:size(temp,2))=temp;\\ \;ii=ii+size(temp,1)\\ end \end{array}$$

### 2.3. Inclusion of the Uncertainty in the ToA

The measurement uncertainties are described by two variables $\delta_{1}$ and $\delta_{2}$, respectively. The former is related to the error introduced by variation in material properties and geometrical tolerances (either numerical or experimental) that may influence the ToA measurement, while the latter is related to the noise introduced by the instrumentation (cross-talk, background noise) and/or environmental conditions (temperature, humidity). It is worth pointing out that the proposed approach is valid for data collected experimentally or simulated numerically. What is included here is the influence of the variability of the measured data on the optimisation process, regardless of how they are generated.

The probabilistic description of the measured ToA in the *i*-th sensor can be expressed as:

(2) $$t_{m_{k}}=t_{c_{k}}+\delta_{1}+\delta_{2}$$

where $t_{m_{k}}$ and $t_{c_{k}}$ are the measured and computed ToA in the *k*-th sensor, respectively. The computed ToA is obtained from the signals simulated by the FE analysis. For simplicity and without loss of generality, $\delta_{1}$ and $\delta_{2}$ are assumed to be two independent Gaussian variables with zero mean and standard deviation $\sigma_{1}$ and $\sigma_{2}$, respectively. If the ToA $\;t_{m_{k}}\;\;\mathrm{for}\;k=1,\ldots ,N_{s}$ ($N_{s}$ being the total number of sensors) are assumed to be independent and identically distributed, the likelihood for any *k* can be expressed as:

(3) $$p\left(t_{m_{k}}|x,\mu_{k},\sigma^{2}\right)=\frac{1}{\sqrt{2\pi \sigma^{2}}}\exp\left[-\frac{1}{2\sigma^{2}}{\left(t_{m_{k}}-t_{c_{k}}\right)}^{2}\right]$$

where x gives the position of the impact, *μ* the mean of the distribution and $\sigma^{2}=\sigma_{1}^{2}+\sigma_{2}^{2}$ means the total variance. The above probabilistic approach is included in the optimisation procedure. The mean value can be assumed to be equal to the computed ToA.

Using Bayes’ theorem, the likelihood can be related to the posterior probability distribution function (PDF) and to the prior distribution as:

(4) $$p\left(x_{i}|t_{m_{k}}\right)=\frac{p\left(t_{m_{k}}|x_{i}\right)\;p\left(x_{i}\right)}{p(t_{m_{k}})}$$

The above relation may be used to determine the location of the impact on the basis of the sensors measurements (for instance, to locate the damage in [17,30]). However, in this work, the Bayes theory is used for sensor optimisation, while the impact detection, as explained in Section 2.1, is performed by an ANN approach.

The optimal sensor configuration can be obtained by solving the following problem:

(5) $$\min_{s\in S}\;p\left[\underset{i\in I}{⋃}\left(x_{i}|e_{i}>e_{i_{T}}\right)\right]$$

where s is a vector collecting the sensor readings, *I* is a set of given impacts each of which is located at $x_{i}$, $e_{i}=\;|x_{i}^{c}-x_{i}^{p}|$ and represents the Euclidean distance between the actual impact location and the predicted impact location. The vector $e_{T}$ represents a threshold value representing the acceptable performance of the SHM system to detect the impact, and for the sake of generality, it can be assumed to be a function of the impact position.

The minimization given in Equation (5) means that the optimal sensor configuration is searched in order to minimize the probability of not detecting an impact occurring at location $x_{i}$. As the impact events are independent, the above problem can be stated as:

(6) $$\frac{1}{C}\min_{s\in S}\;\sum_{i\in I}p\left[\left(x_{i}|e_{i}>e_{T}\right)\right]$$

where *C* is a constant to scale the probability function in the range (0,1), and it can be set equal to the number of impacts in *I*. By applying Bayes’ theorem, we obtain:

(7) $$\min_{s\in S}\;\sum_{i\in I}\left[1-p\left(e_{i}\leq e_{T}|x_{i}\right)\right]p\left(x_{i}\right)=\min_{s\in S}\;\sum_{i\in I}\left[1-PoD_{i}\right]p\left(x_{i}\right)=\min_{s\in S}\;J\left(e_{T}\right)$$

where $PoD_{i}=p\left(e_{i}\leq e_{T}|x_{i}\right)$ is the probability that the impact occurring in $x_{i}$ is identified with an error $e_{i}\leq e_{T}$. The relation (7) is equivalent to the minimization of the Bayes risk as presented in [20] with reference to the damage identification.

If the ToA measurements are assumed to obey Equation (3), then $p\left(e_{i}\leq e_{T}|x_{i}\right)$ is the conditioned marginal probability of $p\left(e_{i}\leq e_{T},x_{i},\sigma^{2}\right)$, i.e.,:

(8) $$\begin{array}{ccc} p\left(e_{i}\leq e_{T}|x_{i}\right) & = & \frac{p\left(e_{i}\leq e_{T},x_{i}\right)}{p(x_{i})}=\frac{\int_{-\infty }^{\infty }p\left(e_{i}\leq e_{T},x_{i},\sigma^{2}\right)d\sigma^{2}}{p(x_{i})}\\ & = & \int_{-\infty }^{\infty }p\left(e_{i}\leq e_{T}|x_{i},\sigma^{2}\right)p\left(\sigma^{2}\right)d\sigma^{2} \end{array}$$

The prior probabilities $p(x_{i})$ and $p(\sigma^{2})$ can be stated as the mathematical representation of the engineering judgement on the unknown variables (the impact location and the variance of the measurement) with the purpose of including any available knowledge prior to the application of the monitoring system in real operation [31]. For composite panels in aeronautics, such terms can be built, for instance, by gathering the impact monitoring data from the fleet.

The integral in Equation (8) can be computed numerically by imposing $\sigma^{2}$ to vary in a fixed pre-defined interval. The $PoD_{i}$ term can be computed by following a Monte Carlo approach and varying the ToA in each sensor in the Gaussian representation. More details are given in Section 2.5.

### 2.4. Sensor Malfunctioning in Optimisation

The Bayesian approach allows a stronger inference between the probability that the impact is identified with the probability that one or more sensors are malfunctioning at the instant of the impact. In this section, a Bayesian sensor optimisation procedure including the probability of sensor failure is presented. The optimal sensor combination can be searched by minimizing the probability of an impact located at $x_{i}$ not being detected (i.e., $e_{i}>e_{T}$) for a given probability of malfunctioning sensors:

(9) $$\min_{s\in S}p\left(x_{i}|M_{s},e_{i}>e_{T}\right)$$

where s is a vector identifying the chosen sensor network from the set S containing all of the possible combinations of $N_{s}$, and $M_{s}$ is a variable giving the number of malfunctioning sensors. The optimal sensor combination can be searched among a chosen set *I* of impacts:

(10) $$\min_{s\in S}\;p\left[\underset{i\in I}{⋃}\left(x_{i}|M_{s},e_{i}>e_{T}\right)\right]$$

As the impacts are all independent events, it can be written:

(11) $$\frac{1}{C}\min_{s\in S}\;\sum_{i\in I}p\left(x_{i}|M_{s},e_{i}>e_{T}\right)$$

where *C* is a constant that normalizes the probability sum, and it can be set equal to the number of impacts in *I* without loss of generality. By applying Bayes’ theorem and assuming that the impact and the sensor malfunctioning are independent events, we have:

(12) $$\min_{s\in S}\;\sum_{i\in I}p\left(e_{i}>e_{T}|x_{i},M_{s}\right)p\left(x_{i}\right)p\left(M_{s}\right)$$

and therefore:

(13) $$\min_{s\in S}\;\sum_{i\in I}\left[1-p\left(e_{i}\leq e_{T}|x_{i},M_{s}\right)\right]p\left(x_{i}\right)p\left(M_{s}\right)$$

Analogously to what has been proposed in the previous subsection, the marginal probability involved in Equation (13) can be expressed as:

(14) $$p\left(e_{i}\leq e_{T}|x_{i},M_{s}\right)=\int_{-\infty }^{\infty }p\left(e_{i}\leq e_{T}|x_{i},\sigma^{2},M_{s}\right)p\left(\sigma^{2}\right)d\sigma^{2}$$

If $PoD_{i,M_{s}}$(x) indicates the probability to detect an impact at $x_{i}$ for a given sensor malfunctioning level $M_{s}$ and a given probability distribution of the ToA measurements, then the minimization process assumes the following expression:

(15) $$\min_{s\in S}\;\sum_{i\in I}\left[1-{\mathrm{PoD}}_{i,M_{s}}\left(x_{i}\right)\right]p\left(x_{i}\right)p\left(M_{s}\right)=\min_{s\in S}\;J\left(e_{T},M_{s}\right)$$

The terms $p\left(x_{i}\right)p\left(M_{s}\right)$ and $p(\sigma^{2})$ represent the prior probabilities of having an impact at location $x_{i}$, having a level $M_{s}$ of sensor malfunctioning and having a certain variance in the ToA distribution, respectively. They are the novelty introduced in this work, by the Bayesian approach to improve the optimisation process by taking into account the experience gathered during the service life of the monitoring system.

Notice that the objective function is scalar for a given sensor combination, provided that $p(M_{s})$ has a pre-assigned spatial distribution in terms of the impact position. Details on the computation of the $PoD_{i,M_{s}}$ term are given in Section 2.5.

### 2.5. Computation of the Probability of Detection

The evaluation of the probability of detection (PoD), is an important factor in the optimisation analysis, since it represents the performance of the impact detection methodology. In order to optimise the sensor locations for detecting each impact scenario with high reliability, the PoD resulting from different sensor combinations must be maximized through an optimisation approach. The procedure outlined in [23] for evaluating the PoD is adopted here with several improvements. An iteration of the optimisation process includes developing an ANN for any sensor combination. To evaluate the performance of each ANN, its response is simulated following the Monte Carlo approach with a certain number of samples. Such samples are reproduced by inclusion of the uncertainty in the ToA and sensor malfunctioning $M_{s}$ and by considering one impact at a time. The error associated with each sample is given by:

(16) $$e_{i}=\sqrt{e_{ix}^{2}+e_{iy}^{2}}$$

which is the Euclidean distance between the predicted location and the actual location of each impact *i* belonging to the test set (all of the available impact scenarios are divided into training, validation and test sets for the development of ANN).

To know which statistical properties best represent the ANN performance, the cumulative distribution function (CDF) must be known. For this reason, the CDF can be built and compared to some analytical distributions. In particular, three analytical CDFs are conceived of by integrating the following three analytical PDFs (a misprint is worth underlining in Equation (15) of [23] in the expression of the Weibull PDF):

(17) $$\begin{array}{ccc} \mathrm{Gaussian}\;\mathrm{PDF} & = & \frac{1}{\sigma \sqrt{2\pi }}exp\left[-\frac{1}{2}{\left(\frac{e_{i}-\mu }{\sigma }\right)}^{2}\right] \end{array}$$

(18) $$\begin{array}{ccc} \mathrm{Lognormal}\;\mathrm{PDF} & = & \frac{1}{e_{i}\sigma_{l}\sqrt{2\pi }}exp\left[-\frac{1}{2}{\left(\frac{\ln e_{i}-\mu_{l}}{\sigma_{l}}\right)}^{2}\right] \end{array}$$

(19) $$\begin{array}{ccc} \mathrm{Weibull}\;\mathrm{PDF} & = & \frac{k}{\lambda^{k}}\;e_{i}^{k-1}\;exp^{-{\left(\frac{e_{i}}{\lambda }\right)}^{k}}\; \end{array}$$

where *μ* and *σ* are the mean and standard deviation of the Gaussian PDF; $\mu_{l}$ and $\sigma_{l}$ are the mean and the standard deviation of lne; *k* and *λ* are the shape parameter and the scale parameter of the Weibull PDF. A parametric study carried out by the authors concluded that the CDF of the error in Equation (16) can be described by any of the above functions. In fact, the quantile–quantile plot shows a discrepancy of a maximum of 5% between the analytical and the numerical CDF.

The analytical function that best describes the probability of detection is identified on the basis of two goodness-of-fit parameters, the normalized root mean square *R* and the coefficient of determination *d*:

(20) $${\mathrm{CDF}}_{best}=\max\left(\frac{1}{R}+d\right)$$

After identifying the best CDF, the probability of detection (either $PoD_{i}$ or $PoD_{i,M_{s}}$) is computed by reading the *y*-value corresponding to $e_{T}$. The present approach stems from [23] to include statistical analysis of the data: the error is investigated by measuring the influence of the uncertainties on the impact location prediction for each impact, whereas in [23], the error is only related to the ANN predictions.

## 3. Validation Examples

There are two validation examples presented in this section: (i) the experimental test on a composite coupon to validate the PoD calculation, including the probabilistic description of the ToA to improve the reliability of the passive detection algorithm; and (ii) a numerical example to assess the application of the proposed Bayesian optimisation methodology on a section of a large composite stiffened panel. For both examples, the recorded/simulated signals from impact events were used for developing ANNs for impact detection. The established ANNs are feedforward multi-layer perceptron (MLP) trained with the ToA of each sensor utilizing the back propagation learning algorithm. The performance of each trained network was then measured by the error function defined as the mean absolute error between the predicted and the actual impact location using the test dataset. For all of the examples presented in this section, the parameters that are required to be defined are as follows:

(a) $N_{cy}$ gives the number of cycles to generate the best ANN;
(b) $P_{sd}=3\sigma$ provides the width of the Gaussian distribution;
(c) $\epsilon_{t}$ gives the strain threshold at which the ToA is computed;
(d) $N_{MC}$ is the number of samples generated in the Monte Carlo approach;
(e) $e_{T}$ provides the measure of the error in the impact localization prediction above which the impact is considered as “not detected”.

### 3.1. Experimental Validation of the Fitness Function: Composite Coupon

**Figure 1:**
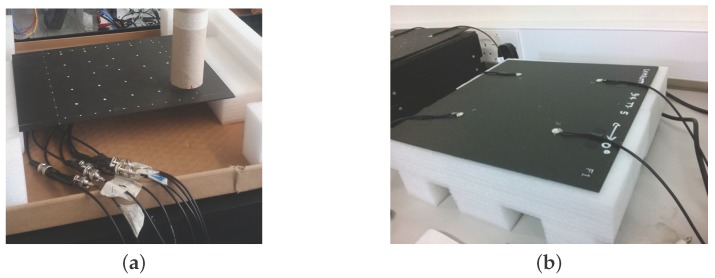
Composite panel subjected to the experimental tests. Top view (**a**); and bottom view with sensors (**b**).

The aim of the first validation exercise is to assess the reliability of the proposed PoD as a realistic measure of the performance of the ANN and to compare it to the case when uncertainty has not been included, in a probabilistic way, in the development of the meta-models as described in [23]. The fitness function, which is minimized in the proposed algorithm, is based on the computed PoD for any sensor combination. The PoD adopted here includes uncertainty in the sensor signals as described in Section 2.3. The proposed PoD (as described in Section 2.5) is examined by conducting the impact experiment on a composite plate of a size of 300 × 200 mm, as shown in Figure 1. Impactors of a diameter of 40 mm and a mass ranging from 2.7 to 19.4 g were dropped from a height of 360 mm on a grid of 20 × 20 mm (representing 100 impacts for each mass) to represent a variety of impact energies on the composite plate. For each impact, the resulting signals at four surface-mounted PZT sensors (see Figure 1b) were measured with a time step of 0.0025 s. The PZT sensors used were PIC 225 Lead Zirconate from PI Ceramics GmbH (Lederhose, Germany), with a diameter of 10 mm and a thickness of 0.5 mm with a wrap-around electrode. The recorded signals’ frequency is in the kHz bandwidth, which is in the working frequency range of the PIC transducer. The signals were first denoised using the wavelet filter and then the ToA measured using a threshold value $\epsilon_{t}$ of 0.08 V.

Once the sensor data are obtained by impacting the panel (depicted in Figure 1), the PoD is computed as in [23] with a noise level of 5% and compared with the PoD computed by the present approach by introducing 5% uncertainty (as described in Section 2.3). The results were obtained by setting $N_{cy}=1000$, $P_{sd}=5\%\mu$ (*μ* was defined in Equation (3)), $e_{T}=$ 40 mm and $N_{MC}=1000$. Furthermore, without loss of generality, the probability of impact occurring at any point on the plate for any sensor malfunctioning, $p(x_{i})$ and $p(M_{s})$ were set to be uniform. The performance of the meta-model without including the uncertainty distribution in the input data provided PoD =89%, whereas the PoD computed by the present procedure resulted in PoD${}_{unc}=67\%$. The detection performance of both procedures was then evaluated with 100 new impacts in order to test the reliability of the computed PoDs. Here, 72 out of 100 impacts were detected with an error e≤40 mm demonstrating that the PoD${}_{unc}$ computed with the present approach (67%) resulted in being more robust. Without including the uncertainty in the experimental data, the PoD measure is overestimated. Therefore, it can be concluded that the proposed fitness function is validated by the experimental results and is a realistic measure of the performance of the meta-models for impact detection. This fitness function will be implemented in the optimisation algorithm in the next step.

**Figure 2:**
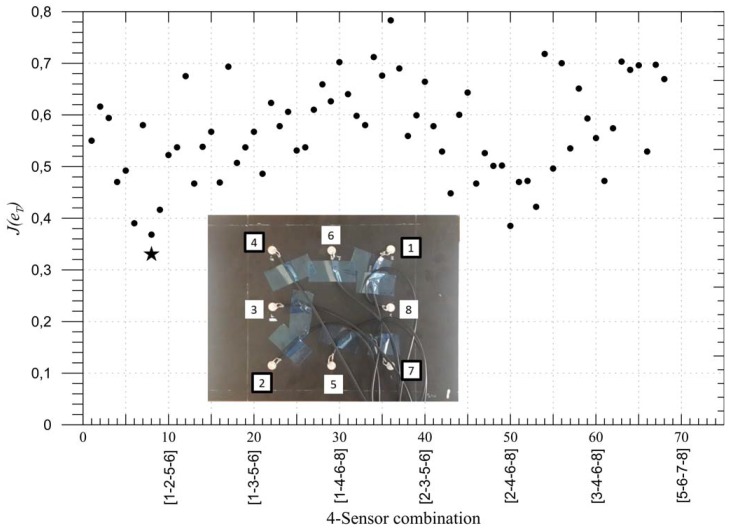
Fitness function $J(e_{T})$ (given by Equation (7)) distribution for all the possible four-sensor combinations. $J{\left(e_{T}\right)}_{best}=0.33\Rightarrow PoD=67\%$.

Now that the proposed fitness function was shown to be a good representation of the performance of the impact detection methodology, the optimisation procedure is applied to the composite panel shown in Figure 1, to find the best four sensor locations out of eight possible locations as the first validation case. As the total number of possible combination was limited (equal to 70), the fitness function $J(e_{T})$ for each combination was calculated and presented in Figure 2. The combinations are numbered along the *x*-axis going from combination no. 1≡ [1-2-3-4] up to combination no. 70≡ [5-6-7-8] with all of the combinations highlighted with a filled circle in the graph. The adopted sensor numbering is shown on the bottom left side of Figure 2. The point representing the optimal value in the graph is represented by a star, whereas the corresponding optimal sensors, that is no. 8≡ [1-2-4-7] corresponding to the four corner sensors as expected, are highlighted by a squared black frame.

### 3.2. Validation of the Bayesian Approach for Sensor Optimisation: Composite Stiffened Panel

**Figure 3:**
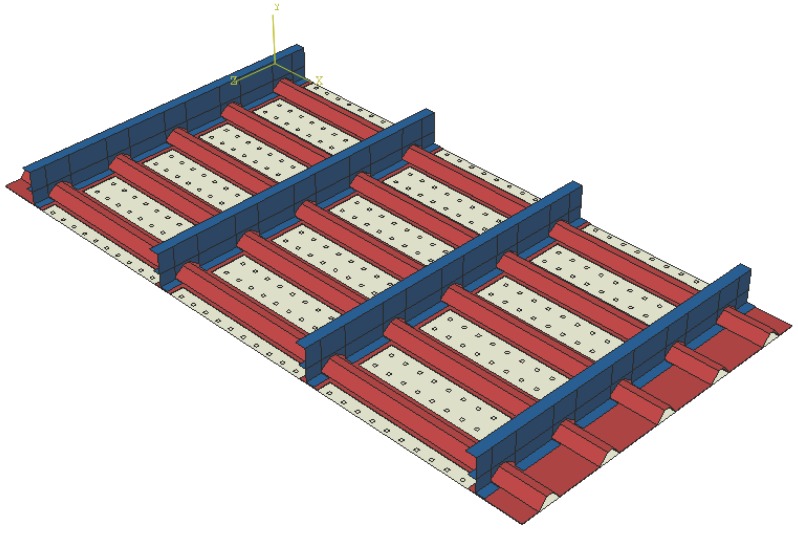
The 2045 × 1070 mm${}^{2}$ stiffened panel with all possible sensor locations.

The second validation example is carried out on a more complex geometry representative of a real structures, i.e., a composite stiffened panel made of unidirectional and woven carbon/epoxy; see Figure 3. A non-linear FE model of the panel (2045 mm by 1070 mm) was developed in the commercial software ABAQUS FEA by Simulia and used to simulate impacts at various locations. The model was meshed using 2 mm general purpose shell elements. More details on the numerical modelling of the impact events can be found in [10].

A total of 1265 impacts of different masses (a large mass and a small mass simulating a tool drop and a hail impact), velocity and locations were carried out to develop a library of all possible impact scenarios on the stiffened panel. The generated ultrasound waves from the impacts were recorded at 300 possible sensor locations, 50 mm apart, shown in Figure 3 by small rectangles.

**Figure 4:**
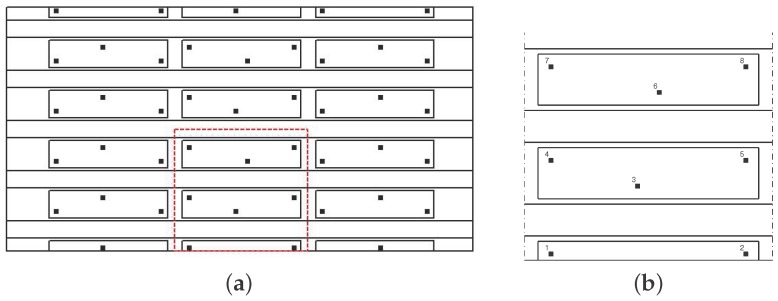
(**a**) Main panel with the one-sixth portion extraction; (**b**) one-sixth portion with eight possible sensor locations.

This optimisation example intends to comparing the performance of the proposed procedure for computing the probability of detection without sensor malfunctioning, that is $PoD_{i}$, and with sensor malfunctioning, that is $PoD_{i,M_{s}}$. As a proof of concept, a portion of the main panel represented in Figure 3 is investigated (see Figure 4), i.e., a portion including one sixth of the entire panel. For each sensor combination, the ANN is built taking into account the impacts occurring on that section.

**Figure 5:**
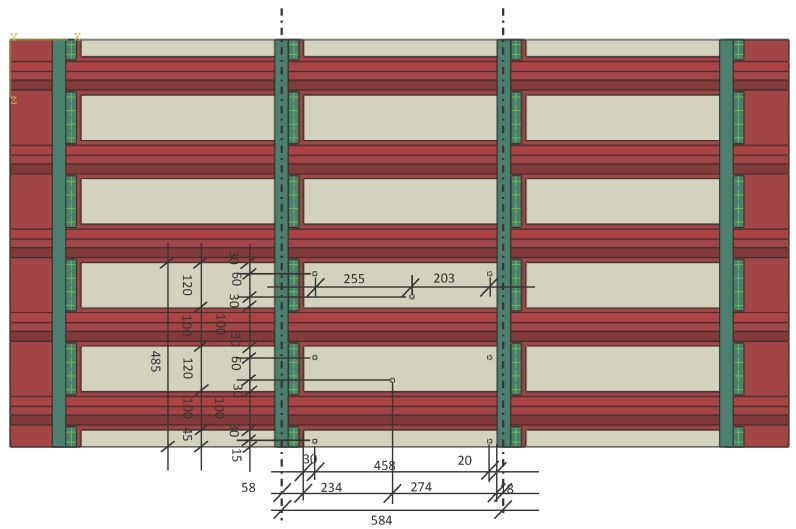
Sensor locations for the section of the panel. The quotes are expressed in millimiters.

Eight possible sensor locations have been proposed as shown in Figure 4b, and the best location of four sensors is searched on the basis of the best ANN performance. The position of the sensors and the size of the panel section are detailed in Figure 5.

The value of the objective function $J(I,M_{s})$ is calculated without sensor malfunctioning and with one and two sensors malfunctioning (Option A of Section 2.2 was considered) following Equations (7) and (15), respectively. The objective function provides a measure of the probability of misdetection; therefore, the lower it is, the better the performance of the ANN is. The efficiency of the sensor combination can be assessed as the complementary value of the objective function, i.e., $100-J(I,M_{s})$, which is referred to as the “performance” of the sensor network and presented in Table 2. The results in Table 2 are obtained by setting $N_{cy}=500$, $P_{sd}=5\%\mu$, $e_{T}=30$ mm, $\epsilon_{t}=10^{-10}$ and $N_{MC}=1000$. Furthermore, without loss of generality, $p(x_{i})$ and $p(M_{s})$ are set as uniform distributions.

As expected, the best sensor locations are the four corners [1-2-7-8], with a performance of 72% that decreases to 61% with one malfunctioning sensor. The reduction in the performance is higher for the sub-optimal networks, as is evident by comparing the performance of the optimal sensor network [1-2-7-8] being 61% with, for instance, a sub-optimal combination [1-4-7-8], which is 37%.

**Figure 6:**
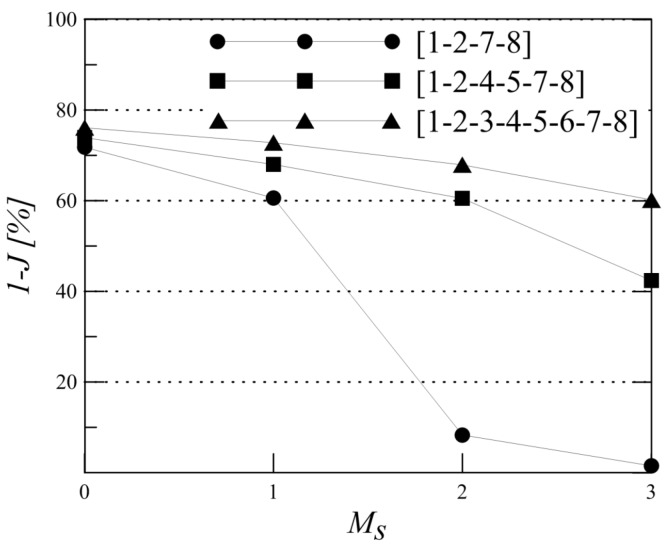
Performance of four-, six- and eight-sensor combinations for increasing sensor malfunctioning $M_{s}$ in the panel represented in Figure 4. $P_{sd}=5\%\mu$ and $e_{T}=30$ mm.

Comparing the performance of the optimal four transducer network with the sub-optimal ones does not show a significant change when sensor malfunctioning is not included. However, when the probability of one sensor malfunctioning is included, the performance of the sub-optimal networks is significantly lower than the optimal network (e.g., 35% compared to 61%). Therefore, the results strongly indicate that including the sensor malfunctioning probability to increase the reliability of the detection does indeed impact the optimal sensor locations and therefore needs to be included in the optimisation strategy. In addition, the results emphasise the importance of increasing the number of transducers to reach a higher reliability of the detection. This is evident from comparing the reduction in performance, due to two sensors malfunctioning, for the four-sensor combination [1-2-7-8], the six-sensor combination [1-2-4-5-7-8] and the eight-sensor combination [1-2-3-4-5-6-7-8], presented in Table 2, and by plotting the performance gradient deterioration against the number of malfunctioning sensors in Figure 6. To reach a required level of performance, the number of sensors (as this is an input) will be increased in an iterative process, starting from four transducers. If the optimal sensor combination will not reach the required PoD level, the number of transducers should be increased and the optimisation algorithm carried out again.

Increasing the tolerance of the acceptable error level $e_{T}$ increases the performance of each sensor combination. For instance, with $e_{T}=50$ mm, the above four-sensor combination [1-2-7-8] (with no malfunctioning) performance raises to 83%, whereas combinations [1-2-4-5-7-8] and [1-2-3-4-5-6-7-8] reach both 86%, indicating that by increasing the number of sensors, the performance does not necessarily improve.

## 4. Optimal Sensor Location for a Composite Stiffened Panel

In the previous section, two examples were presented that validated the proposed fitness function, including uncertainty in the input data (simulating real application) and also accounting for the probability of sensor malfunctioning in the optimisation algorithm. The results concluded that when the sensor malfunctioning is included in the optimisation strategy, the performance of the optimal sensor network is indeed higher than the other sub-optimal combinations.

**Figure 7:**
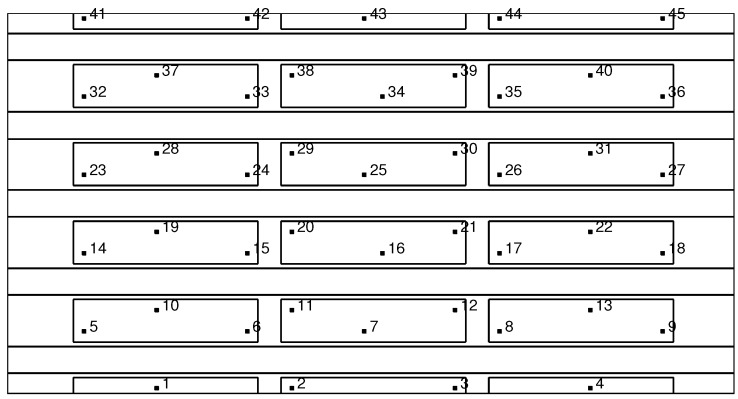
Stiffened panel with forty-five possible sensor locations.

The next step is to assess and validate the proposed strategy by applying it to the full composite stiffened panel depicted in Figure 3. To do so, an optimisation approach based on GA was carried out to find the best four- and five-sensor networks. To reduce the dimensionality and computational cost of the problem, a total of forty-five possible sensor locations has been identified for this example; see Figure 7.

First, the optimisation is carried out to find the best four-sensor combination, with the following parameters: $N_{cy}=500$, $P_{sd}=5\%\mu$, $\epsilon_{t}=10^{-10}$, $N_{MC}=1000$, $e_{T}=50$ mm. An initial population of N=200 individuals is set. At each iteration of the (GA), a new population of *N* individuals is generated from the previous one by retrieving $n_{c}=4$ elite individuals, mutating with 20% probability and by cross-over of 80% of the parents (where 80 is the percentage out of $N-n_{c}$). Furthermore, without loss of generality, $p(x_{i})$ and $p(M_{s})$ are set as uniform distributions.

The optimal sensor combination and its performance are listed in Table 3. It can be seen that the best performance is achieved when the sensors are placed at the corners of the plate [1-4-41-45], maximizing the coverage area. To validate that the chosen sensor combination is indeed the optimal one, its performance has been compared to similar sensor combinations with similar coverage areas. The results in Table 3 show that the performances of the sensor networks are very similar, but the optimal one is slightly higher. Furthermore, as expected, their performance deteriorates quickly with increasing the probability of malfunctioning sensors.

**Figure 8:**
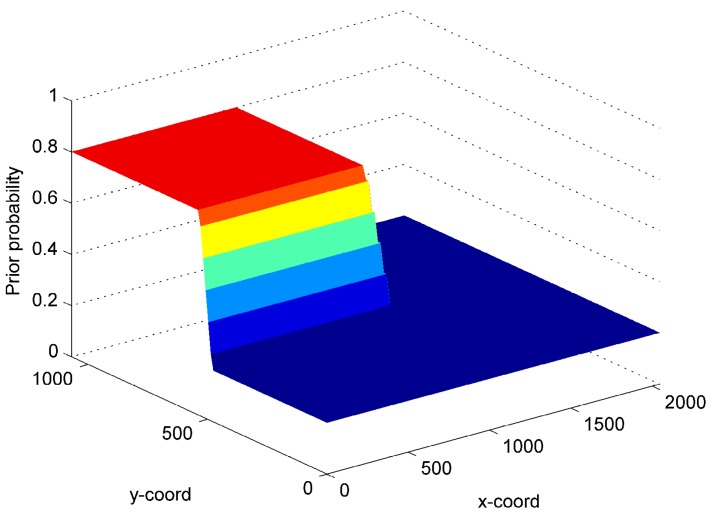
Non-uniform prior probability p(x) considered in the example depicted in Figure 9(b). *x*- and *y*- coordinates in millimeters.

**Figure 9:**
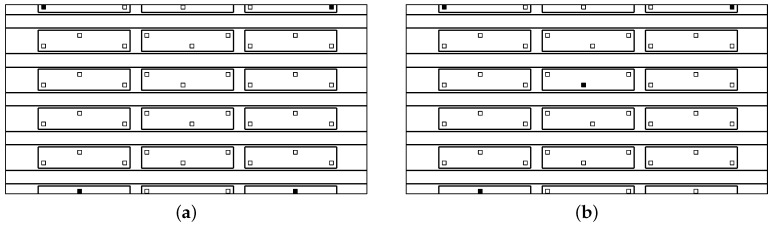
Best four-sensor combination. Uniform probability (**a**); non-uniform probability (**b**).

So far, in the examples presented, the probability of impact occurrence (i.e., p(x)) on the structure is assumed to be uniform. However, a real structure, such as aircraft panels, does not have the same probability of impact occurrence everywhere. One of the advantages of the proposed Bayesian approach is that it allows the user to include a non-uniform probability distribution of impact in the optimisation approach. This feature is presented on the stiffened panel by comparing the best four-sensor combinations with uniform and non-uniform probability distributions p(x). The non-uniform probability is defined by assuming that it is more likely to have impacts near the top-left corner zone (in an area equal to the half of the entire panel), as presented in Figure 8. The optimal solutions are depicted in Figure 9.

For the uniform distribution of p(x), the minimization process locates the sensors close to the corners (see Figure 9a). On the contrary, the optimal solution with the non-uniform prior probability has one sensor closer to the area with higher probability of impact (see Figure 9b). The other three sensors remain in the corner position as the lower probability is set equal to 0.2. Decreasing such a probability would move the three corner sensors towards the top-left area. The performances of the two (uniform and non-uniform) optimal combinations provided in Figure 9 are listed in Table 4 (for $P_{sd}=10\%\mu$, $\epsilon_{t}=10^{-10}$, $e_{T}=45$ mm).

The last example searches the optimal locations for a five-sensor network, from all of the possible locations in the panel, as shown in Figure 7. The optimisation procedure is carried out with reference to a detection level of $e_{T}=20$ mm, a Gaussian distribution of ToA provided by $P_{sd}=10\%\mu$ and uniform probability p(x). All of the remaining parameters are identical to the previous example.

**Figure 10:**
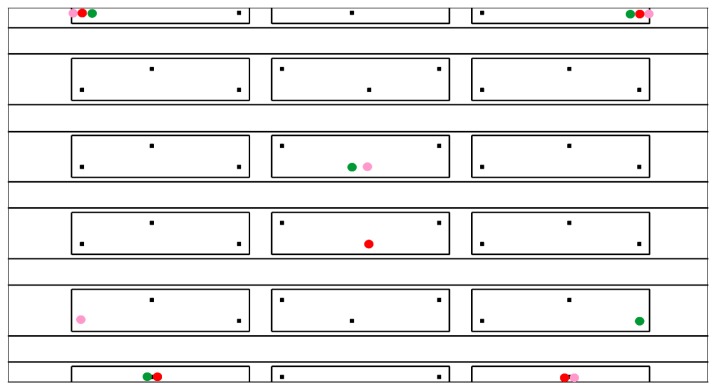
Best five-sensor combinations. Sensors with the same color belong to the same combination.

**Figure 11:**
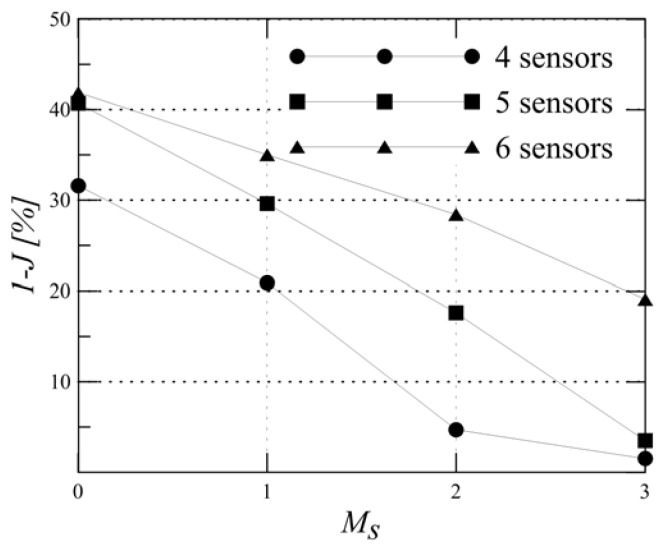
Performance of four-, five- and six-sensor combinations for increasing sensor malfunctioning $M_{s}$ in the panel represented in Figure 7. $P_{sd}=10\%\mu$ and $e_{T}=20$ mm.

The optimal and sub-optimal combinations are provided in Figure 10, where they are characterized by different colours, that is red (optimal), green and pink (sub-optimal). The value of 1−J provided by the optimal combination is 41%. The performance can be improved by reducing the error threshold $e_{T}$; for instance, with $e_{T}=50$ mm, the performance increases to 84%. The comparison between combinations with different numbers of sensors (see Figure 11) shows that the performance versus the number of sensor malfunctioning $M_{s}$ deteriorates more quickly with a lower number of sensors.

## 5. Conclusions and Future Work

A Bayesian probabilistic approach for sensor optimisation for impact detection, which includes uncertainties arising from the application of the SHM system to real structures, was reported. The approach includes variances in sensor measurement due to environmental and operational conditions, the probability of one or more sensor malfunctioning and the non-uniform probability of impact occurrence. The proposed approach was shown to increase the probability of detection and the reliability of the passive SHM system when subjected to complex structures, such as a composite stiffened panel under operational conditions. In such a Bayesian framework, an objective function was established in order to compute the sensor combination that can predict the impact location, with the highest probability. The impact detection methodology adopted in this work is the meta-models based on ANN. However, the fitness function required as input to the optimisation strategy can be based on any impact detection technique. Both experimental and numerical examples have been carried out to assess the applicability of the proposed optimisation strategy to realistic composite structures with complex geometries.

The main conclusions drawn from the proposed optimisation strategy are:

(i) by introducing a probabilistic measure of ToA as input to the ANN, a more realistic PoD is measured, which was validated by experimental results;
(ii) including sensor malfunctioning in the optimisation procedure (to increase the reliability of the SHM system) results in a different optimal sensor location. In addition, the number of malfunctioning sensors influences their optimal placement. Therefore, it is very important that the designers of the SHM system define the rate and the probability of sensor malfunctioning a priori and include them in the optimisation algorithm;
(iii) the proposed methodology allows the definition of the non-uniform probability of impact occurrence. It was shown that the optimal sensor location changes when different probabilities of impact occurrence are defined for the same structure, and it increases the overall probability of impact detection when considered in the optimisation strategy.

## Figures and Tables

**Table 1:** Global percentages of impacts by zones of the aircraft [22].

Zones	Fuselage	Wings	Nose	Cone and Rear	Doors	Passenger	Cargo Door
	Sections			Fuselage		Door Surroundings	Surroundings
Impact (%)	7	13	7	5	15	31	22

**Table 2:** Performance of different sensor combinations for the panel depicted in Figure 4 without ($M_{s}=0$) and with ($M_{s}=1,2$) sensor malfunctioning.

Sensor Combination	Performance (%),	Performance (%),	Performance (%),
	$M_{s}=0$	$M_{s}=1$	$M_{s}=2$
[1-2-7-8] (optimal)	72	61	8
[1-3-7-8]	65	35	-
[1-4-7-8]	63	37	-
[1-5-7-8]	69	51	-
[1-2-4-5-7-8]	74	68	60
[1-2-3-4-5-6-7-8]	76	73	68

**Table 3:** Comparison of the best four-sensor combinations for the panel depicted in Figure 7.

Sensor Combination	Performance (%),	Performance (%),	Performance (%),
	$M_{s}=0$	$M_{s}=1$	$M_{s}=2$
[1-4-41-45] (optimal)	83.1	74.1	9.6
[1-9-41-45]	82.8	73.8	10.2
[4-5-41-45]	82.8	73.8	9.3

**Table 4:** Comparison of network performance for uniform prior probability (Performance${}_{upp}$) and variable prior probability (Performance${}_{vpp}$).

Sensor Combination	Performance${}_{\mathrm{upp}}\left(\%\right)$	Performance${}_{\mathrm{vpp}}\left(\%\right)$
[1-4-41-45] (optimal with uniform probability)	70	65
[1-25-41-45] (optimal with non-uniform probability)	62	72
